# Domestication and the storage starch biosynthesis pathway: signatures of selection from a whole sorghum genome sequencing strategy

**DOI:** 10.1111/pbi.12578

**Published:** 2016-06-11

**Authors:** Bradley C. Campbell, Edward K. Gilding, Emma S. Mace, Shuaishuai Tai, Yongfu Tao, Peter J. Prentis, Pauline Thomelin, David R. Jordan, Ian D. Godwin

**Affiliations:** ^1^ School of Agriculture and Food SciencesThe University of QueenslandBrisbaneQldAustralia; ^2^ Department of Agriculture and Fisheries (DAF)WarwickQldAustralia; ^3^ BGI‐ShenzhenShenzhenChina; ^4^ Queensland Alliance for Agriculture and Food InnovationThe University of QueenslandWarwickQldAustralia; ^5^ Science and Engineering FacultyQueensland University of Technology (QUT)BrisbaneQldAustralia; ^6^ Australian Centre for Plant Functional GenomicsGlen OsmondSAAustralia

**Keywords:** Sorghum (*Sorghum bicolor*), domestication, starch synthesis, whole‐genome sequencing, selection, metabolic pathway

## Abstract

Next‐generation sequencing of complete genomes has given researchers unprecedented levels of information to study the multifaceted evolutionary changes that have shaped elite plant germplasm. In conjunction with population genetic analytical techniques and detailed online databases, we can more accurately capture the effects of domestication on entire biological pathways of agronomic importance. In this study, we explore the genetic diversity and signatures of selection in all predicted gene models of the storage starch synthesis pathway of *Sorghum bicolor*, utilizing a diversity panel containing lines categorized as either ‘Landraces’ or ‘Wild and Weedy’ genotypes. Amongst a total of 114 genes involved in starch synthesis, 71 had at least a single signal of purifying selection and 62 a signal of balancing selection and others a mix of both. This included key genes such as *STARCH PHOSPHORYLASE 2* (*SbPHO2*, under balancing selection), *PULLULANASE* (*SbPUL*, under balancing selection) and ADP‐glucose pyrophosphorylases (*SHRUNKEN2*,* SbSH2* under purifying selection). Effectively, many genes within the primary starch synthesis pathway had a clear reduction in nucleotide diversity between the Landraces and wild and weedy lines indicating that the ancestral effects of domestication are still clearly identifiable. There was evidence of the positional rate variation within the well‐characterized primary starch synthesis pathway of sorghum, particularly in the Landraces, whereby low evolutionary rates upstream and high rates downstream in the metabolic pathway were expected. This observation did not extend to the wild and weedy lines or the minor starch synthesis pathways.

## Introduction

The study of the evolution of metabolic pathways is fundamental to understanding evolutionary change as well as key events in the domestication process (Fraser *et al*., 2002; Ramsay *et al*., 2009). By examining the evolution of genes in an integrated metabolic pathway, we can gain some understanding of the complex forces that are shaping domesticated germplasm and question whether the differential selection acting upon certain loci conforms to previous studies that sought to explain how the structure of such pathways affects evolutionary rate (Clotault *et al*., 2012; Livingstone and Anderson, 2009; Ramsay *et al*., 2009; Rausher *et al*., 2008). Positional rate variation (PRV) is the theory that low evolutionary rates upstream and high rates downstream upon genes within a metabolic pathway is expected as a consequence of purifying selection. This increase in selective constraint is believed to occur because the consequence of a nonsynonymous mutation within a gene at a key branch point higher up in the synthesis process could lead to major pleiotropic effects that result in no useful end products (Rausher *et al*., 1999). This was the case within the plant biosynthetic pathways of isoprene (Sharkey *et al*., 2005), anthocyanin (Lu and Rausher, 2003; Rausher *et al*., 1999, 2008), carotenoid (Clotault *et al*., 2012; Livingstone and Anderson, 2009) and terpenoid synthesis (Ramsay *et al*., 2009). But does this model hold for the critical process of storage starch synthesis? Unlike other pathways, the starch synthesis pathway (SSP) is not a simple unidirectional pathway but also contains several alternative branches resulting in starch as the terminal product and has to support respiration and other key processes. Similarly, the SSP contains a series of catabolic genes that encode enzymes that work in reverse. Cereal grains are the single most important source of calories in the world, predominantly in the form of starch, and comprise as much as 80% of the calorific intake for some of the poorest countries (WHO, 2003). The SSP is one of the most well‐characterized pathways in plant science and has undergone strong selection during domestication (James *et al*., 2003; Tetlow *et al*., 2004; Whitt *et al*., 2002; Zeeman *et al*., 2010). With the exception of soya bean, all of the top 10 most important human food crops (cereals; roots; tubers; plantains) are eaten primarily for their starch content. Hence, the SSP is essential to human food security (http://faostat3.fao.org/browse/rankings/commodities_by_regions/E). Starch is composed of two D‐glucose homopolymers: amylose and amylopectin (Smith, 2001). Amylose consists predominantly of α‐1,4 linked glucosyl monomers with low–moderate branching, while amylopectin, the more abundant polymer, contains highly branched (through α‐1,6 glucosyl bonds) linear chains of various lengths (Smith, 2001).

**Figure 1:**
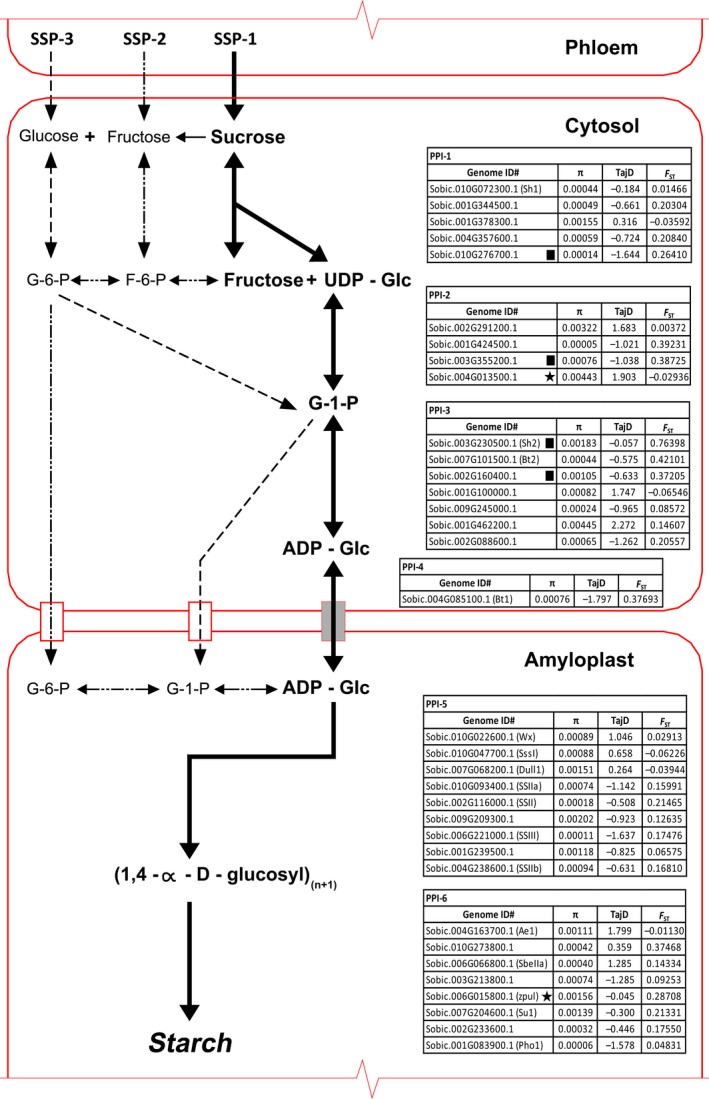
Plant (monocot) starch synthesis. Diagram of the reactions involved in starch synthesis. Arrows represent single or groups of enzymes that convert one metabolite to another. The primary starch synthesis pathway from sucrose to starch is represented by bold arrows (SSP‐1). Other pathways examined in this study from fructose to starch (SSP‐2) and glucose to starch (SSP‐3) in sink tissues are represented by 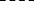 and 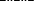, respectively. Expressed genes for the primary pathway have been sorted according to the Pathway Pleiotropy Index (PPI; Ramsay *et al*., 2009), with CDS values calculated across all lines for π, Tajima's D and *F*_ST_ (Landraces vs wild and weedy). Genes under purifying selection are represented with a (■) and under balancing selection with a (★).

Starch synthesis requires the function of a number of vital enzymes, primarily ADP‐glucose pyrophosphorylases (AGP; *SHRUNKEN2, Sh2* and *BRITTLE ENDOSPERM2*,* Bt2*), starch synthases (*STARTCH SYNTHASE‐I*,* SSI*;* SUGARY2*,* su2*;* STARCH SYNTHASE‐IIb*,* SSIIb*,* DULL 1*,* du1* and *WAXY*,* wx1*) and the starch‐branching (SBE; *STARCH‐BRANCHING ENZYME 1*,* sbe1* and *AMYLOSE EXTENDER 1*,* Ae1*) and debranching (DBE; *SUGARY1*,* su1* and *PULLULANASE*,* zpu1*) enzymes (James *et al*., 2003; Tetlow *et al*., 2004). Several isoforms of these enzymes exist, leading to a highly complex synthesis process. Starch production begins with the conversion of glucose 1‐phosphate to ADP‐glucose utilizing the enzyme ADP‐glucose pyrophosphorylase. Starch synthase enzymes then link the ADP‐glucose via a α‐1,4 glycosidic bond to an emerging chain of glucose residues, releasing ADP and generating amylose. The creation of branched amylopectin takes place when starch‐branching enzymes introduce α‐1,6 glycosidic bonds between these chains, some of which are later removed by starch debranching enzymes (Figure 1) (James *et al*., 2003; Smith, 2001).

The majority of studies investigating the evolution of metabolic pathways in higher plants have included only small numbers of genes that are well characterized, frequently only with partial sequence availability and/or confined to well‐studied species such as Arabidopsis, maize and rice (Li *et al*., 2012; Slotte *et al*., 2011; Whitt *et al*., 2002).

With the advent of next‐generation sequencing (NGS), all predicted gene models in the genome can be sequenced in a single run. The NGS strategy facilitates the evolutionary analysis of entire metabolic pathways by utilizing population genetic techniques (Wright and Gaut, 2005; Yu *et al*., 2011) and metabolic pathway data available via online databases such as www.gramene.org/pathways. Furthermore, these techniques will be integral for the discovery of key candidate genes of importance to breeding programmes, especially in the light of the terabytes of data that now require analysis and the fact remarkably little is known about the nature of biochemical pathways, mutations and genes with respect to adaptive evolution (Wright and Gaut, 2005).

Utilizing the important cereal species sorghum (*Sorghum bicolor*) as a model, we examine the genetic history of starch at a systems biology level through the lens of energy storage in sorghum grain. This seminal work provides a framework for understanding the changes brought about by evolution and domestication. Changes in expression and specific enzyme functionality are levels of complexity beyond the purview of this manuscript, which aims to determine how genetic changes could have adapted genes for the purpose of starch storage in the grain.

Several studies on sorghum have utilized population genetic analysis techniques to study the impacts of domestication, linkage disequilibrium and sequence polymorphism (de Alencar Figueiredo *et al*., 2008; Casa *et al*., 2005, 2006; Frere *et al*., 2011; Hamblin *et al*., 2004, 2006, 2007; Mace *et al*., 2013; Morris *et al*., 2013). Of these, only Hamblin *et al*. (2007), de Alencar Figueiredo *et al*. (2008) and Frere *et al*. (2011) specifically examined genes involved in the SSP. Hamblin *et al*. (2007) explored sequence and linkage disequilibrium (LD) variation in partial sequence in 15 genes in the SSP, while de Alencar Figueiredo *et al*. (2008) investigated sequence diversity of partial sequence in six candidate sorghum homologs of known maize SSP genes for grain quality (*Sh2*,* Bt2*,* SssI*,* Wx*,* Ae1* and *opaque‐2*). A more recent study by Frere *et al*. (2011) sought to detect evidence of artificial selection upon seed storage proteins and partial sequence of three starch biosynthesis loci (*SSIIa*, sbe*I* and *sbpul*).

The main objective of this study was to identify whether the SSP is under strong selective pressure utilizing population genetic measures focussed upon complete gene model sequences from a diverse set of sorghum germplasm. Further, it was questioned whether selective pressure acts differentially upon genes depending on their position within the metabolic pathway.

## Results

### The starch biosynthesis pathway and patterns of expression

None of the 114 unique starch synthesis genes analysed showed any significant difference (posterior probability of being differentially expressed (PPDE) = ≥0.95) in expression between solar noon and solar midnight. Some gene functions prominent in the dark cycle are involved with protective activities against catabolism, whereas during the light cycle we observe the expression of gene functions involved in protein maturation, carbon metabolism and auxin signal transduction (Table S4). Of note is the increase in expression of the alpha‐amylase inhibitor (Sobic.002G077500.1) and protease inhibitor (Sobic.002G078800.1) indicating that transcription might not be the level at which regulation is occurring in some genes of the grain. However, our transcriptome is primarily a tool to validate expression of genes in the pathway (Table S4).

For SSP‐1, five genes were associated with PPI‐1 (sucrose + UDP to UDP‐D‐glucose + D‐fructose). *SUCROSE SYNTHASE‐I* (*SbSSI*, Sobic.010G072300.1) was the most abundant SSP‐1 transcript with a day average FPKM of 362.48 (Table 1 and Figure 1). The metabolism of UDP‐D‐glucose (PPI‐2) into α‐D‐glucose 1‐phosphate involved four genes, with the key enzyme responsible, UDP–glucose pyrophosphorylase (Sobic.002G291200.1) reaching a day average FPKM of 383.08, which was expressed ≈26× greater than the next most expressed gene (Sobic.001G424500.2).

**Table 1:**
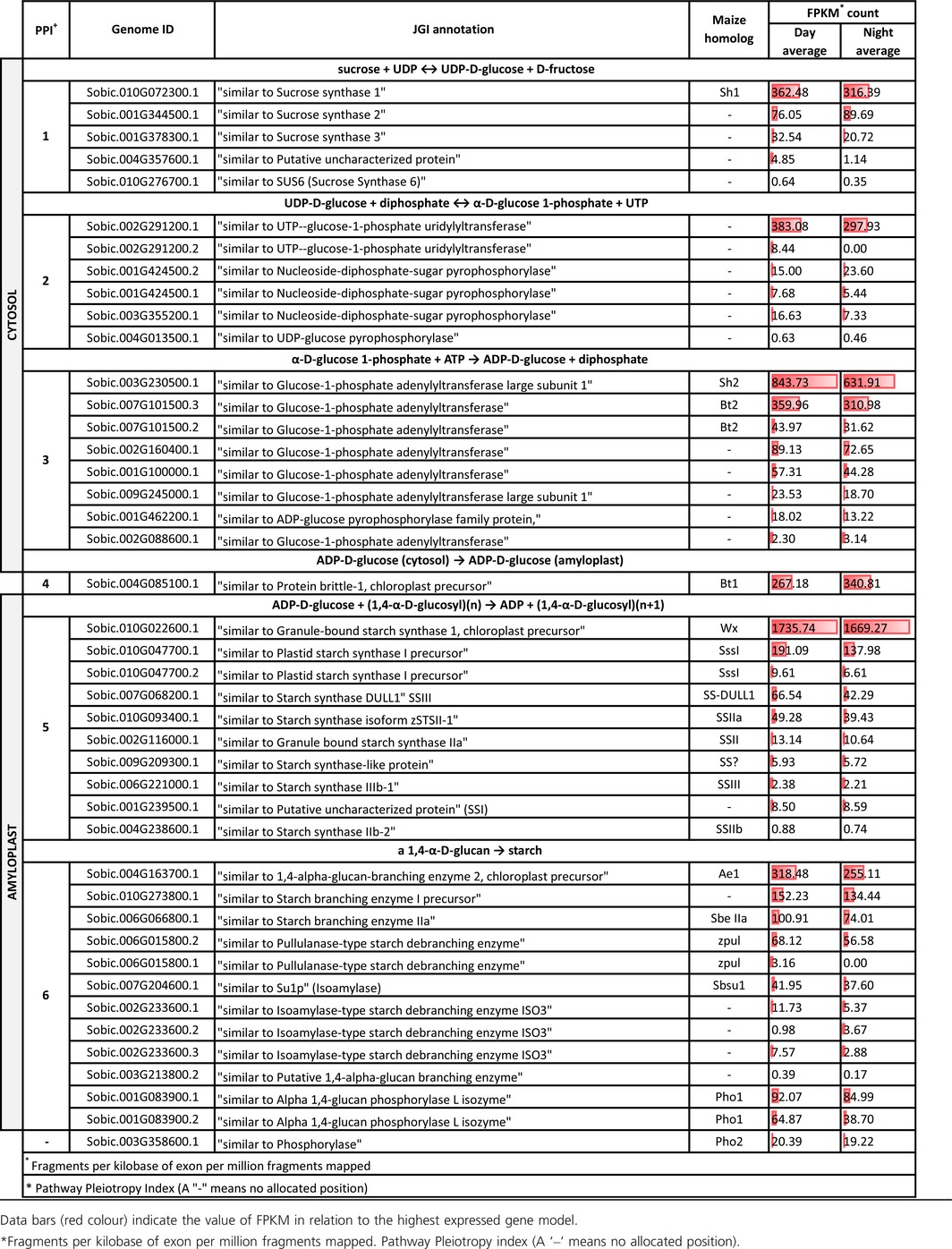
Fragments per kilobase of exon per million fragments mapped (FPKM) of genes involved in the primary starch synthesis pathway from immature grain 16 days post anthesis, sampled at solar midday and midnight

Eight AGP‐related isozyme genes showed expression at PPI‐3 (α‐D‐glucose 1‐phosphate + ATP to ADP‐D‐glucose + diphosphate). The genes with homology to the maize homologs *Sh2* (large subunit) and *Bt2* (small subunit) were Sobic.003G230500.1 and Sobic.007G101500.3, respectively. Of these eight genes, the genes with the highest levels of expression were the homologues of *Sh2* (large subunit) and *Bt2* (small subunit) with FPKM of 843.73 and 359.96, respectively (Table 1 and Figure 1). At PPI‐4, free ADP‐D‐glucose is then actively transported through the ADP‐glucose transporter (*BRITTLE‐1, SbBT1*; Sobic.004G085100.1) into the amyloplast (Figure 1). This gene had a day average FPKM of 267.18.

PPI‐5, the critical stage of converting ADP‐D‐glucose + (1,4‐α‐D‐glucosyl)(n) to linear chains of amylose and amylopectin, demonstrated nine SS loci showing expression within immature sorghum grain. The SS with the greatest expression was Sobic.010G022600.1 (*SbWX*) with a day average FPKM of 1735.74, which was ≈ 9× more expressed than the next closest SS (Sobic.010G047700.1 or *SbSSI* at 191.09) (Tables 1, S2 and S3).

The final stage in the SSP‐1 (PPI‐6) involves the creation of branched amylopectin via the action of SBEs and DBEs. Of the four isozymes of SBEs identified, *SbSBEIIb* or *Ae1* (Sobic.004G163700.1) was the most highly expressed with an FPKM of 318.48. Amongst the DBEs, *SBPUL* (Sobic.006G015800.2; 68.12) was more highly expressed than the isoamylase genes. Expression of the two genes functionally related to starch phosphorylase revealed that Sobic.001G083900.1 (Alpha 1,4‐glucan phosphorylase L isozyme; *SbPHO1*) was more highly expressed with a day average FPKM of 92.07 with transcriptomic data revealing an expressed alternative transcript (Table 1 and Figure 1).

SSP‐2 and SSP‐3 initiate the conversion of sucrose + H_2_O to D‐fructose + α‐D‐glucose (PPI‐1). Six genes were expressed at this point, with the highest expression observed for beta‐fructofuranosidase (neutral invertase) with day average FPKM of 10.67 (Sobic.K041200.1) (Tables S2 and S3).

At PPI‐2, for both SSP‐2 and SSP‐3, hexokinase enzymes convert either D‐fructose + ATP to fructose‐6‐phosphate + ADP in the case of SSP‐2, or to convert α‐D‐glucose + ATP to α‐D‐glucose 6‐phosphate + ADP in the case of SSP‐3. Fifty‐two genes were expressed at PPI‐2, with several homologs of hexokinase present, in particular, the gene hexokinase‐8 with a day average FPKM of 67.79 (Table S2).

The conversion of fructose‐6‐phosphate to β‐D‐glucose‐6‐phosphate (PPI‐3) of SSP‐2, involves 22 genes. This included glucose‐6‐phosphate isomerase (Sobic.002G230600.1; FPKM 11.29), with the remaining genes involved with sugar transport or related to integral membrane proteins (Table S2).

At PPI‐5 of SSP‐2 and PPI‐3 of SSP‐3, the conversion of α‐D‐glucose 6‐phosphate to α‐D‐glucose 1‐phosphate takes place, with three genes containing phosphoglucomutase or phosphomannomutase functionality identified, chief of which is Sobic.001G116500.1, which had a day average FPKM of 58.45. After this point in both SSP routes, they proceed through the pathway steps already described for SSP‐1 leading to starch synthesis (Tables S2 and S3).

### Ka/Ks ratio and the correlation with pathway position

The Ka/Ks ratio can be used to deduce the direction and extent of selection, with a ratio greater than one denoting positive selection, less than one indicating purifying (stabilizing) selection and a ratio equal to one inferring neutral or no selection. Up to 85.7% of genes in the SSP‐1 (all lines) had a Ka/Ks ratio of less than 1, with 18 of these genes identified as being significantly different from 1 (≤0.05), and only one gene had a Ka/Ks greater than 1 (1.63; sucrose synthase‐6; Sobic.010G276700.1).

**Figure 2:**
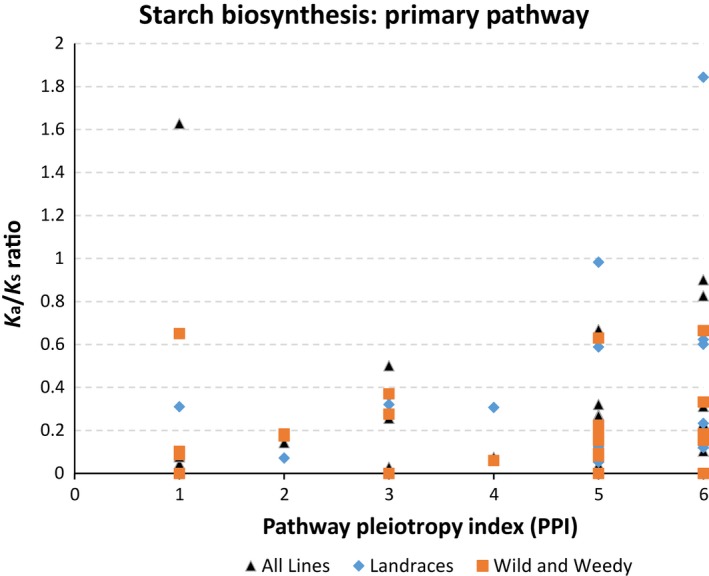
The relationship between K_a_/K_s_ and Pathway Pleiotropy Index (PPI; Ramsay *et al*., 2009) for the Primary Starch Biosynthesis Pathway. K_a_/K_s_ ratio values’ comparisons are shown between sorghum groups ‘all lines’, ‘Landraces’ and ‘wild and weedy’.

This trend was clear across groups and the two other SSP routes, which had 84% of Ka/Ks ratio values <1 (39 genes which were significantly different from 1, ≤0.05) and 85% of Ka/Ks ratio values <1 (33 genes which were significantly different to 1, ≤0.05), for SSP‐2 and 3, respectively. This indicated that the SSP pathway is under the influence of strong purifying selection (Figure 2). No genes had a Ka/Ks significantly greater than 1.

Kendall's τ rank correlation coefficient revealed there was a clear correlation between PPI and Ka/Ks ratio for the primary SSP of the Landraces (τ value = 0.338, *P*‐value ≤0.023). The correlation for SSP‐1 remains significant (τ value = 0.298, *P*‐value ≤0.05) even when the highest Ka/Ks value (1.84) is removed. The correlation between PPI and Ka/Ks ratio for all the lines combined (τ value = 0.202, *P*‐value = 0.145) and the wild and weedy lines (τ value = 0.201, *P*‐value = 0.203) was not significant. However, the removal of the highest Ka/Ks value (1.62) from the combined analysis of all genotypes did result in a significant correlation (τ value = 0.284, *P*‐value ≤0.044) (Figure 2). There was no significant correlation between any derivation of the Ka/Ks ratio data and PPI for either of the other SSPs.

### Sequence diversity in cultivated and wild sorghum

**Figure 3:**
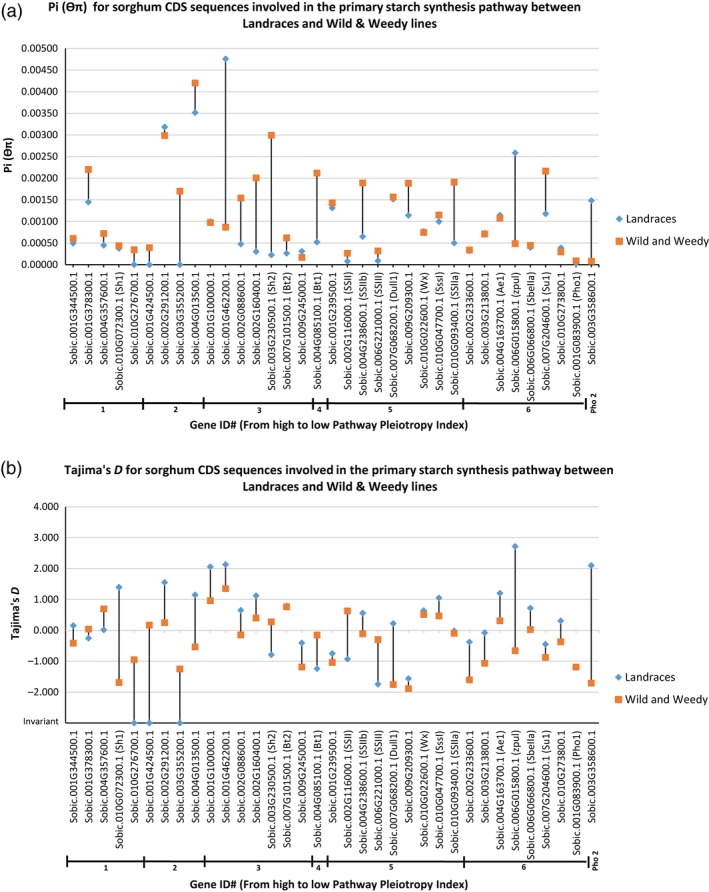
Comparison between Landraces and wild and weedy sorghum lines for CDS sequence of genes involved in the Primary Starch Synthesis Pathway for (a) nucleotide diversity (θ_π_) and (b) Tajima's D. Tajima's D values for data bars marked with ‘Invariant’ are purely for graphical display only and in no way represent that actual value. Genes are sorted according to Pathway Pleiotropy Index (PPI; Ramsay *et al*., 2009).

**Figure 4:**
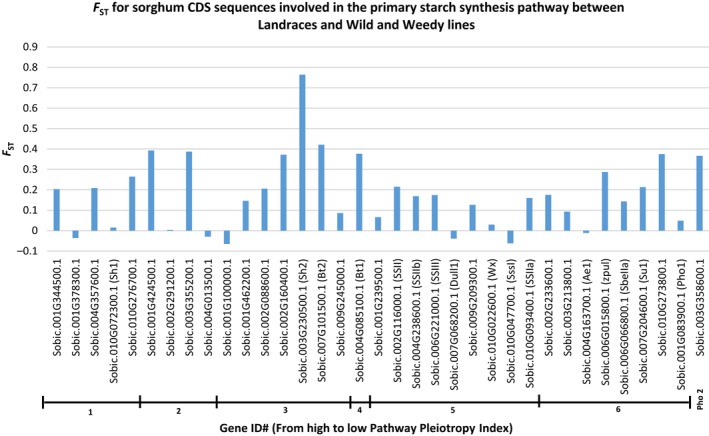
The *F*_ST_ of CDS sequence and genes of the primary Starch Synthesis Pathway of sorghum. *F*_ST_ value comparisons are shown between sorghum groups ‘Landraces’ and ‘wild and weedy’. Genes are sorted according to Pathway Pleiotropy Index (PPI; Ramsay *et al*., 2009).

The primary sequence level comparisons made in this study were focussed at the coding sequence (CDS) level. The mean level of θ_π_ for the genes in the SSP‐1 was 0.00108, with a range of 5.49E‐05 for a sugar pyrophosphorylase (Sobic.001G424500.1) involved in conversion of UDP‐D‐glucose to α‐D‐glucose 1‐phosphate up to 0.00445 for an ADP‐glucose pyrophosphorylase (Sobic.001G462200.1) (Figure 1). For SSP‐2, the mean θ_π_ was 0.00125, ranging from 0 for a sugar transporter (Sobic.008G111100.1) involved in conversion of fructose‐6‐phosphate to β‐D‐glucose‐6‐phosphate up to 0.00678 for a cell wall invertase (Sobic.004G166700.1) (Table S6). Mean θ_π_ for SSP‐3 was 0.00137 ranging from 5.90E‐05 for *SbPHO1* up to 0.00678 for the cell wall invertase in SSP‐2 (Sobic.004G166700.1) (Table S7). Clear reductions in nucleotide diversity (θ_π_) and high levels of genetic differentiation (*F*_ST_) were observed in the genes in the primary SSP between the Landraces and the wild and weedy genotypes, based both at the whole CDS and nucleotide levels (Figures 3a and 4), with a ~1.4‐fold reduction in mean θ_π_ between Landraces (0.0009933) and wild and weedy lines (0.00119). Amongst the genes which showed a substantial decline in θ_π_ between Landraces and wild and weedy genotypes were key enzymes at pathway position branch points including phosphoglucomutase (91.8%; Sobic.001G116500.1), glucose‐6‐phosphate isomerase (95.4%; Sobic.002G230600.1), various hexose transporters (90.5%; e.g. Sobic.003G084000.1) and glycosyltransferases (98%; Sobic.003G094600.1), as well as several AGPases such as *SbSH2* with a 92.4% reduction; *SbBT1* with a 75.4% reduction; and *SbSSIIa* (Sobic.010G093400.1) and *SbSSIIb* (Sobic.004G238600.1) with a 73.8% and 65.5% reduction, respectively (Tables S5 and S6).

Conversely, several genes have shown an increase in θ_π_ between Landraces and wild and weedy lines, with ten genes in SSP‐1, twenty‐eight genes in SSP‐2 and twenty‐six genes in SSP‐3. Chief amongst these genes for increased θ_π_ were *SbPHO2* (94.6%) and the DBE *SbPUL* (81.2%) (Table S5).

Two genes, encoding SEC15 (involved in vesicle trafficking) and a shikimate metabolic gene, respectively, were within the lower 5% of the distribution of nucleotide diversity, and they are not well characterized (Sobic.003G158700.1 and Sobic.010G066700.1). However, both are associated with the conversion of D‐fructose to fructose‐6‐phosphate and had θ_π_ values of 8.73E‐05 and 0.000173, respectively (Tables S6 and S7). Two genes have become invariant within Landraces since domestication, and these were a nucleoside‐diphosphate‐sugar pyrophosphorylase (Sobic.003G355200.1) and an uncharacterized gene (Sobic.010G066700.1) involved in the conversion of D‐fructose to fructose‐6‐phosphate. Conversely, five genes were within the upper 95% of the empirical distribution of θ_π_ in the Landraces, including an alcohol dehydrogenase (0.0103; Sobic.010G071900.1), AGPase (0.00977; Sobic.001G462200.1), glycosyltransferase (0.00778; Sobic.010G144400.1), neutral invertase (0.00694; Sobic.004G166700.1) and a UDP‐glucose pyrophosphorylase (0.00748; Sobic.004G013500.1) (Tables S5–S7).

Four genes had high levels of genetic differentiation (*F*_ST_) between the Landraces and wild and weedy genotypes: *SBSH2* (0.764), hexokinase (0.63; Sobic.009G203500.1), hexose (*HEX6*) transporter (0.628; Sobic.001G297600.1) and a high‐affinity potassium transporter (0.627; Sobic.006G061300.1) (Figure 4; Tables S5–S7).

### Tests of selection at the whole gene and base pair level

**Figure 5:**
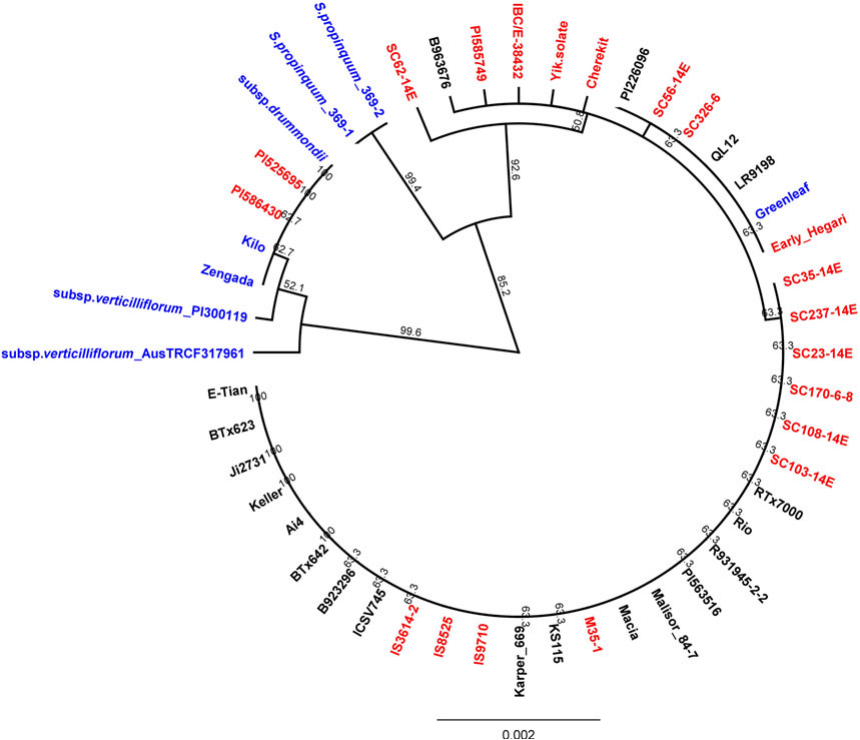
Phylogenetic tree for the whole gene sequence of the large subunit ADP‐glucose pyrophosphorylase (*Sh2*; Sobic.003G230500.1) constructed utilizing the Unweighted Pair Group Method with Arithmetic mean (UPGMA) algorithm. Improved Inbred lines are labelled in ‘Black’; Landraces are labelled in ‘Red’; and Wild and Weedy lines are labelled in ‘Blue’.

**Figure 6:**
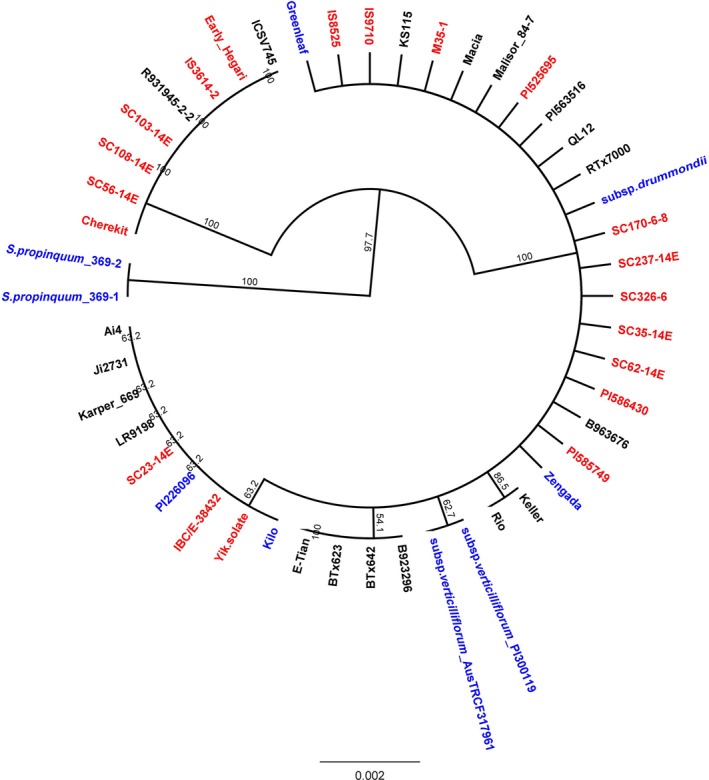
Phylogenetic tree for the whole gene sequence of starch debranching enzyme pullulanase (Sobic.006G015800.1) constructed utilizing the Unweighted Pair Group Method with Arithmetic mean (UPGMA) algorithm. Improved Inbred lines are labelled in ‘Black’; Landraces are labelled in ‘Red’; and Wild and Weedy lines are labelled in ‘Blue’.

In total, 71 genes were identified with signatures of purifying selection versus 62 genes with a signal of balancing selection, including *SbSH2* (purifying selection) and *SbPUL* (balancing selection) (Figures 5 and 6). These genes included several important classes such as AGPases, SS, SBE, DBE as well as genes that code for proteins at key branch points, for example sucrose synthase (Sobic.001G344500.1). Of these, 43 genes also had coding regions under both purifying and balancing selection, including hexokinases, sugar transporters, AGPases (e.g. *SbSH2*), SBE (e.g. *SbAE1*) and DBE (e.g. *SbPUL*) enzymes (Tables S9 and S10).

Fifteen sites in phosphoglucomutase (Sobic.001G116500.1) showed signatures of purifying selection, all synonymous, whereas only a single site was identified within 28 genes such as *SbAE1* (Table S9). In contrast, the number of codons identified as under balancing selection per gene ranged from 18 in both an alcohol dehydrogenase (seven nonsynonymous sites; Sobic.010G071900.1) and glycosyltransferase (nine nonsynonymous sites; Sobic.010G144400.1) through to only single sites identified within 23 genes such as SS *SbDU1* (Sobic.007G068200.1) (Table S10).

Within well‐characterized genes, particularly those involved in the SSP after the conversion of glucose 1‐phosphate to ADP‐glucose, nonsynonymous SNPs were largely occurring in coding regions outside of known protein domains and/or substrate binding sites. For example, *SbSH2* has four nonconservative mutations under purifying selection, which results in changes to amino acid polarity and possibly in protein folding.

Six genes in the SSP were identified with signatures of purifying selection at the gene level. Four of these genes were detected within the primary SSP and include a nucleoside‐diphosphate‐sugar pyrophosphorylase (Sobic.003G355200.1), two AGPases including *SbSH2* (Sobic.003G230500.1 and Sobic.002G160400.1) and a sucrose synthase (Sobic.010G276700.1). The remaining genes included a hexokinase (Sobic.009G203500.1) and uncharacterized gene (Sobic.010G066700.1), which are located at PPI‐2 of both the SSP‐2 and SSP‐3 pathways and play a part in the conversion of D‐fructose into fructose‐6‐phosphate or the conversion of α‐D‐glucose into α‐D‐glucose 6‐phosphate (Figure 4).

Nine genes were identified with signatures of balancing selection. The genes *SbPHO2* and *SbPUL* were identified at the lower PPI of the SSP. The other genes with signatures of balancing selection were detected higher up in the SSP, the majority at PPI‐2 of both the SSP‐2 and SSP‐3 pathways, and included glycosyltransferase and alcohol dehydrogenase functions (Sobic.010G144400.1; Sobic.002G195700.1; Sobic.005G225500.1; Sobic.010G216700.1; Sobic.010G071900.1; Sobic.006G211900.1).

Utilizing the mlHKA test to validate gene level domestication candidates for patterns of genetic variation (positive or balancing selection) showed that a model of directional selection best explained the patterns of polymorphism to divergence of the five candidate genes under purifying selection relative to 34 neutral loci (mean log likelihood ratio test statistic = 283.8; *P* < 0.0001 for all comparisons; Table S11). Likewise, a model of balancing selection best explained the patterns of genetic variation in the nine candidate genes under balancing selection (mean log likelihood ratio test statistic = 310.13; *P* < 0.0001 for all comparisons; Table S11).

Matches between orthologs from maize identified as domestication candidates (Hufford *et al.,* 2012; Jiao *et al*., 2012) and sorghum (gene and base pair level) revealed eight orthologous genes involved in starch biosynthesis with evidence of selection signatures in both maize and sorghum (Tables S9 and S10), including genes such as SS (*SbSSIIa*), isoamylase (Sobic.007G204600.1), *SbSBEI* (Sobic.010G273800.1) and hexokinase‐8 (Sobic.003G035500.1). For rice (He *et al*., 2011; Huang *et al*., 2012; Xu *et al*., 2011), a hexose sugar transporter (*HEX6*) (Sobic.001G297600.1) and alcohol dehydrogenase (Sobic.006G211900.1) were identified with signatures of selection in both sorghum and rice.

The potential for genetic hitchhiking or selective sweeps, whereby strong positive selection on a favourable new mutation can lead to neighbouring chromosomal regions becoming fixed over time, was also examined. Fifty‐two genes were analysed for selective sweeps based on their signatures of selection, association with grain quality traits or presence within SSP‐1 (Table S8). Of these, 48 loci had evidence of selective sweeps with genes identified as being under selection or fixed (invariant) within sorghum groupings. The functional classes of genes under selection were broad and variable, with some of known importance to agronomic traits or domestication. These included *SbGhd7* (Sobic.001G298400.1), in the vicinity of a hexose transporter (Sobic.001G297600.1), which may confer differences in photoperiod sensitivity and flowering times. *SbRd* (Sobic.003G230900.1), located in the vicinity of the AGP (*SbSH2*), is thought to be involved in proanthocyanidin synthesis or pericarp colour. *Psy1* (Sobic.003G230900.1), which belongs to the phytoene synthase family of genes and is linked to carotenoid content and grain colour, was located near a sucrose synthase‐like gene (Sobic.010G276700.1). And finally, *qSH1* (Sobic.003G356200.1), situated near a GDP‐mannose pyrophosphorylase (Sobic.003G355200.1), involved in conversion of UDP‐D‐glucose to α‐D‐glucose 1‐phosphate, which codes for a BEL1‐type homeobox‐containing protein involved in seed shattering in rice. Of all the SSP genes under selection, a UDP‐glucose pyrophosphorylase (Sobic.004G013500.1; balancing selection) had the highest number of genes (6) clustered (four balancing and two purifying) around its genomic location, with flanking genes 0.073 and 0.063 Mb either side. The protein function of the genes surrounding the UDP‐glucose pyrophosphorylase included a peptidyl‐prolyl cis‐trans isomerase (Sobic.004G013400.1) and putative glutamate receptor (Sobic.004G013300.1).

To measure any deviation from the standard coalescent model, which assumes at its most basic level, that no recombination, natural selection or gene flow is occurring within a population, the statistical test Tajima's D was employed. The results revealed a mean Tajima's D value for genes in the primary SSP of −0.144, ranging from −1.80 to 2.27 for *SbBT1* and AGPase (Sobic.001G462200.1), respectively (Figure 3b; Table S5). Likewise, the mean level of Tajima's D for SSP‐2 and SSP‐3 was −0.332 and −0.254, respectively (Tables S6 and S7). The lowest Tajima's D value for these pathways was for a sugar transporter (Sobic.008G111100.1), which was invariant, and the highest an alcohol dehydrogenase (Sobic.006G211900.1) with a value of 3.12. The mean level of Tajima's D for all the pathways deviates from the standard neutral model (< or >1), with the magnitude of the statistic trending towards purifying selection.

Several genes had a clear reduction in Tajima's D between the Landraces and wild and weedy genotypes, including granule bound starch synthase II (−0.93 vs 0.63; *SbGBSSII,* Sobic.002G116000.1), *SbSSIII* (−1.74 vs −0.30; Sobic.006G221000.1), *SbBT1* (−1.24 vs −0.15), *SbSH2* (−0.79 vs 0.27) and sucrose synthase‐3 (−0.25 vs 0.04; Sobic.001G378300.1) (Figure 3b; Table S5). Genes specific to SSP‐2 and SSP‐3 with a clear reduction in Tajima's D between the groups included key hexokinases such as hexokinase‐8 (−0.50 vs 0.12; Sobic.003G035500.1) and hexokinase‐3 (−0.87 vs 0.54; Sobic.003G421200.1) as well as sugar transporters such as this mannitol transporter (invariant vs 0.17; Sobic.001G469600.1) and hexose transporter (−1.19 vs −0.30; Sobic.003G084000.1) (Tables S6 and S7). Twenty‐six genes showed a clear increase in Tajima's D in the Landrace genotypes in comparison with the wild and weedy genotypes in SSP‐1. This pattern was also observed for 59 genes in SSP‐2 and 52 genes in SSP‐3 and included such genes as *SbPHO2*,* SbPUL*, sucrose synthase‐1 (Sobic.010G072300.1), cathepsin B‐like cysteine protease (Sobic.002G315800.1) and glycosyltransferase (Sobic.010G144400.1) (Tables S5–S7).

## Discussion

Selective constraints were found to be unevenly distributed within the primary SSP of sorghum. The level of purifying selection generally correlates with the hierarchical position of the genes, with upstream genes invariably being the most constrained (Waxman and Peck, 1998). This observation has also been made within unidirectional plant metabolic pathways such as isoprene (Sharkey *et al*., 2005), anthocyanin (Lu and Rausher, 2003; Rausher *et al*., 1999, 2008), carotenoid (Clotault *et al*., 2012; Livingstone and Anderson, 2009) and terpenoid biosynthesis (Ramsay *et al*., 2009). Several of the gene families involved in starch synthesis at the lower pathway position levels (from the conversion of glucose 1‐phosphate to ADP‐glucose) encode key enzymes responsible for the diversity of different starch qualities and quantities for a multitude of end uses. Hence, human selection is certain to play a role in their fixation or diversification. Many of these genes are the products of ancient whole‐genome duplications, for example AGPase, SS (Paterson *et al*., 2004), which have led to subfunctionalization as well as the formation of protein complexes (Tetlow *et al*., 2008). Particular homologs of these genes that undergo higher evolutionary rates (Li *et al*., 2012), in some cases, asymmetrically (Corbi *et al*., 2011; Georgelis *et al*., 2007) were observed. In contrast, purifying selection largely affected genes higher up in the SSP. This may explain why PRV can occur within the primary SSP regardless of the bidirectional and/or cyclic action of many of the downstream enzymes.

However, this result does not mirror the observations made in rice and its wild ancestor (*O. rufipogon*). Yu *et al*. (2011) did not detect any correlation between the levels or patterns of diversity and pathway position within the SSP. Our study extended analysis of pathway position prior to the conversion of glucose 1‐phosphate to ADP‐glucose, which may explain these differences.

There were 47 genes present for the conversion of D‐fructose to fructose‐6‐phosphate, which may explain our observation that PRV was not occurring for the minor routes leading to starch synthesis (SSP‐2 & SSP‐3). Without more experimental evidence, it is impossible to rule candidate genes in or out for many of the stages higher up in SSP and this result should be re‐examined in future when such data become available.

Distinct signatures of selection amongst genes involved in the SSP during domestication and evident under both natural and human selection conditions reiterate the findings that the genes involved in the SSP are remarkably conserved amongst grasses (Li *et al*., 2012). This changing flux in purifying selection revealed several genes to have notable reductions in nucleotide diversity including isozymes of ADP‐glucose pyrophosphorylase (e.g. *SbSH2*), *SbBT1, SbSSIIa*, as well as phosphoglucomutase, glucose‐6‐phosphate isomerise, hexose transporters and glycosyltransferases.

In concordance with Hamblin *et al*. (2007), there were clear divergences in the level of selection between certain sorghum genes and their ortholog in maize (da Fonseca *et al*., 2015; Whitt *et al*., 2002) and rice genes (Yu *et al*., 2011). The most prominent examples were the increased nucleotide diversity of SBEIIb (*Ae1*) and isoamylase (*su1*). Correspondingly, the reduction in nucleotide divergence for *Wx*,* Bt2* and *Sh2* compared with maize was also detected (de Alencar Figueiredo *et al*., 2008; Hamblin *et al*., 2007). This underlined that the selection acting upon particular genes within the SSP through domestication, essentially leading to larger grain size, higher starch content and different starch structural properties, can take alternative paths.

The central importance of starch production to higher plants as a means to store high‐density energy in the form of carbohydrate, for use with essential growth and metabolic requirements, is already well documented (Tetlow, 2011). Likewise, the fundamental importance of cereal starch for human needs and industrial uses (Burrell, 2003) explains why signatures of purifying selection have been found not only in genes present both upstream and downstream within the starch synthesis pathway of the Landraces but also within the wild and weedy lines. The interfertility within *Sorghum* also means that many wild relatives can readily cross‐pollinate with improved germplasm (e.g. Sagnard *et al*., 2011).

This study identified as many as 90 genes under some form of selection. Moreover, 43 upstream genes have showed evidence of differential selection, whereby specific genes have several base pairs showing signatures of purifying selection and other regions showing balancing selection.

The phosphoglucomutase and the AGPase subunit *Sh2* genes encode enzymes which work sequentially in the SSP via generation of Glc‐1‐P from Glc‐6‐P and then by Glc‐1‐P and ATP to generate ADPGlc and inorganic pyrophosphate (Tetlow, 2011). Both genes were under purifying selection. While all enzymes of a pathway contribute to the control of metabolic flux into a product, strong evidence exists, particularly in potato tubers, that both of these enzymes make a significant contribution to the flux control conversion of sucrose to starch (Geigenberger *et al*., 2004). While the contributions of these enzymes to the control of flux into starch have not been quantified in cereal endosperm, mutants of AGPase do lead to a low‐starch phenotype (Stark *et al*., 1992) while overexpression provides the essential substrate needed for greater starch content and seed size in domesticated cereals (Tuncel and Okita, 2013). Previously identified as being a target for domestication in sorghum (de Alencar Figueiredo *et al*., 2008; Hamblin *et al*., 2007), certain allelic variants of *Sh2* and *Bt2*, which work synergistically as a protein complex, have been shown to be associated with yield in sorghum (de Alencar Figueiredo *et al*., 2010). Interestingly, the enzyme believed to make the greatest contribution to flux control during starch accumulation (Geigenberger *et al*., 2004), the ADP‐glucose transporter (Bt1), which is the primary route for transport of ADP‐glucose into the amyloplast, did not generate the strongest signals of purifying selection. However, clear reductions in nucleotide diversity and Tajima's D between Landraces and wild and weedy genotypes were observed, combined with purifying selection at the codon level of three sites (two of which were nonsynonymous) and balancing selection at two sites, suggesting that domestication has made identifiable alterations to this key protein.

Nine genes were under balancing selection at the whole gene level within the SSP, including such genes as *SbPHO2*,* SbPUL,* UDP‐glucose pyrophosphorylase (Sobic.004G013500.1) and various uncharacterized glycosyltransferase and alcohol dehydrogenase genes. The functional role of pullulanase and its influence upon starch structural properties is not well understood. Knockout mutations for this gene in maize and rice has not revealed clear grain phenotypes (Dinges *et al*., 2003; Fujita *et al*., 2009), but a distinct relationship between a particular pullulanase allelic variant and increased sorghum grain digestibility has been demonstrated (Gilding *et al*., 2013). While no clear connection between the high digestibility allele type (*SbPUL‐RA*) and end use has been established, the well‐defined partition in the gene tree between genotypes carrying this allele and the others carrying its less digestible counterpart (*SbPUL‐GD*) could be a reason for the balancing selection acting upon this gene (Gilding *et al*., 2013).

UDP‐glucose pyrophosphorylase is a key enzyme for carbohydrate metabolism, catalysing the reversible production of UDP‐glucose and pyrophosphate from Glc 1‐P and UTP. The UDP‐glucose resulting from this reaction also serves as a key substrate in the biosynthesis of cell wall polysaccharides (Gibeaut, 2000). While it has been shown that reducing UGPase activity via mutation has little effect upon carbohydrate content under controlled conditions (Meng *et al*., 2009; Zrenner *et al*., 1995), Arabidopsis mutants had significantly decreased fitness under field conditions evidenced by a 50% reduction in seed set than wild‐type plants (Meng *et al*., 2009). A proposed mechanism for the reduced fitness was the observation in mutant studies of rice, that the UGPase enzyme was essential for pollen viability (Chen *et al*., 2007; Woo *et al*., 2008) as well as the need for starch during pollen development (Mu *et al*., 2009). While UGPase is believed not to be a rate‐limiting enzyme in SSPs, it is possible that balancing selection may be responding to the interacting selective pressures of larger seed size as well as overall yield, which is part of the domestication syndrome, or its essential role in providing UDP‐glucose for cell wall biogenesis means that in actively dividing cells, such as developing endosperm, its activity would likely be an important yield determining factor at early stages of development during active cell division prior to grain filling. Conversely, other isozymes of this gene family are shown to be under purifying selection in sorghum (Sobic.003G355200.1), illustrating the complex dynamics of selection on this pathway.

Understanding why balancing selection has maintained polymorphism in starch phosphorylase (*SbPHO2*) is less clear. Starch phosphorylase catalyses the reversible transfer of glucosyl units from glucose‐1‐phosphate to the nonreducing end of α‐1‐4 linked glucan chains and can serve in either a degradative or synthetic role depending on the relative concentration of its substrates (Tetlow, 2011). Two major isoforms of starch phosphorylase enzyme are known to exist in higher plants and have been termed plastidic (chloroplast/amyloplast, *Pho1*) and cytosolic (*Pho2*), respectively (Nakano and Fukui, 1986). In terms of starch synthesis, the exact action of *Pho1* is unclear; however, several groups have shown that its gene expression correlates with biosynthesis in developing endosperm of maize (Yu *et al*., 2001) and rice (Satoh *et al*., 2008) as well as in this study. Further, Satoh *et al*. (2008) demonstrated via *Pho1* rice mutants that this gene plays a central role in starch synthesis, content and structure during low temperatures. There is no known role for *Pho2* in starch metabolism as starch granules are bound within the chloroplast/amyloplasts, and its relative expression compared with *Pho1* was lower in this study and the Morokoshi database (Makita *et al*., 2015). Hypothetical roles for *Pho2* extend to the possible degradation of reserve starch in plant organs in which the starch‐containing cells have lost their compartmental integrity (Buchner *et al*., 1996; Schupp and Ziegler, 2004) and/or metabolizing products of starch degradation as well as regulating cytosolic Glc‐1‐P levels (Rathore *et al*., 2009).

Sugar transporters are expressed during sorghum grain fill, yet, due to a lack of experimental information, we were unable to integrate those genes into the primary SSP and had to rely on the information detailed in the SorghumCyc online database. In fact, the full list of genes analysed in this study may not be exhaustive or conversely and contain members which do not significantly participate in starch synthesis; for example, several genes had FPKM expression close to zero, particularly within PPI‐2 of SSP‐2 and SSP‐3. Bioinformatic analysis of differential expression within the RNA‐Seq data also showed a lack of statistically significant differences in diurnal expression for any of the SSP genes. SBEs have been reported to be diurnally expressed (Mutisya *et al*., 2009), and our data show that the SBEs are ≈30% down‐regulated during the night; however, the two approaches are not directly comparable since our analysis is quantitative versus the semiquantitative analysis conducted by Mutisya *et al*. (2009). Ultimately, relative differences in transcript abundance do not provide any information regarding enzyme activity which is more reliant on the amount of enzyme protein synthesized and the extent to which this does or does not turn over within the cell.

The allelic diversity present within a number of starch synthesis loci was substantial amongst the diverse sorghum set analysed in this study and included nonsynonymous SNPs that may lead to altered protein function, providing an opportunity to conduct further starch structural studies and enhance our understanding of this complex synthesis process. This would be particularly useful for the study of the SBEs, which may possess allelic variants that could generate high amylose starches which are not currently available in sorghum and are valuable for their human health attributes and utility in the manufacture of biomaterials. Given the levels of allelic diversity observed within our restricted sampling of wild relatives (both *verticilliflorum* and *propinquum*), and the guinea‐margaritiferums, germplasm from these sources represent diverse and valuable sources of new genetic variation.

## Materials and methods

### Plant materials

Resequencing data from accessions of *Sorghum bicolor*, representing all races of cultivated sorghum, in addition to its progenitors and *S. propinquum* were studied (Mace *et al*., 2013) (Table S1). The NGS data achieved an average final effective mapping depth of ≈22× per line (16× to 45×) with a SNP calling accuracy of 99.85% and 99.72% as validated via targeted sequencing and whole‐genome *de novo* assembly of representative lines, respectively.

### Sample preparation for RNA‐Seq and subsequent analysis

The RNA‐Seq experiment generated transcriptomic data from grain heads sampled 16 days after anthesis, within the critical window for peak grain fill. In brief, samples were run through a pipeline consisting of the RNA extraction protocol of Li and Trick (2005) followed by standard sample preparation for RNA‐seq on the Ion Torrent platform (Thermo Fisher Scientific, Waltham, MA). RSEM, a software package for determining expression counts for transcripts, was employed to derive the FPKM values used and fed into a differential expression analysis using EBSeq (Detailed summary; Method S1).

### Identification of starch biosynthesis genes

A multistage process was employed to identify a list of high‐confidence starch biosynthesis genes. The first stage involved utilizing the SorghumCyc online database (http://pathway.gramene.org/gramene/sorghumcyc.shtml), to identify the complete set of genes believed to be components of the SSP based on known or predicted gene identification, using published resources or homology and/or hidden Markov models (HMM) calculations. There are several SSP routes, and this study mainly focused on the primary route in monocots (SSP‐1), which initiates with the breakdown of sucrose into UDP‐D‐glucose + D‐fructose and continues with the conversion of UDP‐D‐glucose into ADP‐D‐glucose, which is primarily transported by an ADP‐glucose transporter (*Bt1*) into the amyloplast for final starch synthesis (Figure 1). SSP schematics via metabolism of D‐fructose (SSP‐2) and α‐D‐glucose (SSP‐3) were also analysed, but due to the dynamic process of starch synthesis these pathways are not the only possible routes (Figure 1). In the second stage, this list of genes was then compared to genes shown to be expressed in developing endosperm via the RNA‐Seq experiment, identifying 35 genes in SSP‐1, 106 genes in SSP‐2 and 86 genes in SSP‐3.

In addition to the genes confirmed to be expressed via the RNA‐Seq data, a further 3 genes were added to the analysis list, based on published data that suggested they played a prominent role in starch synthesis. These genes were *PULLULANASE* (*SbPUL,* Sobic.006G015800.1), a starch DBE with certain allelic variants shown to contribute to sorghum grain *in vitro* digestibility (Gilding *et al*., 2013), and two genes consisting of the plastidial *STARCH PHOSPHORYLASE 1* (*SbPHO1*, Sobic.001G083900.1) and cytosolic *STARCH PHOSPHORYLASE 2* (*SbPHO2*, Sobic.003G358600.1), which are functionally related to starch phosphorylase (alpha 1,4‐glucan phosphorylase) and have been shown to play a prominent role in starch metabolism (Blennow *et al*., 2002) and is functionally expressed (Ohdan *et al*., 2005). All genes were expressed in immature sorghum grain (Tables 1, S2 and S3). Genomic sequence of the 114 identified starch metabolism genes in sorghum was extracted from the NGS data (Mace *et al*., 2013) with a focus on Landraces and wild and weedy groups (Table S1). These sorghum genes were also contrasted with orthologs from maize (Hufford *et al.,* 2012; Jiao *et al*., 2012) and rice (He *et al*., 2011; Huang *et al*., 2012; Xu *et al*., 2011) that have been characterized as under selection.

### Assignation of pathway position and assessments of selection

Pathway position for the 114 genes found within the 3 SSP routes was assigned to their location based on the Pathway Pleiotropy Index (PPI) (Ramsay *et al*., 2009), which ascribes ‘single’ or ‘groups’ of enzymes that act between pathway branch points to their set position in the metabolic pathway and numbers them from upstream to downstream positions. Selection of PPI to assess PRV was made due to its superiority to track changes in pleiotropy along a pathway and avoid the influence of factors that can mitigate the role of pathway structure upon evolutionary rate variation such as gene duplication.

The degree of selective pressure acting on protein coding regions was measured utilizing the Ka/Ks ratio. Calculating Ka and Ks substitution rates was conducted using the software package KaKs_Calculator1.2 (MYN method) (Zhang *et al*., 2006). Correlation between Ka/Ks ratio and PPI was calculated utilizing Kendall's τ rank correlation coefficient (Ramsay *et al*., 2009).

### Population genetic analyses

Nucleotide diversity per site (θ_π_) (Nei, 1987), Watterson's estimator (θ_W_) (Watterson, 1975) and the neutrality test Tajima's D (Tajima, 1989) were estimated for (CDS and gene) each of the 114 starch synthesis genes (Mace *et al*., 2013). These parameters were directly calculated for each genetic component using a BioPerl module and an in‐house perl script. *F*_ST_ was calculated on the same genetic components to measure population differentiation using an alternative BioPerl module (Mace *et al*., 2013).

Genes with signatures of purifying selection were identified by the criteria utilized in Mace *et al*. (2014): θπ and θw in the lower 5% of the empirical distribution in the descendent population, *F*_ST_ values >95% of the empirical distribution and negative Tajima's D. Likewise, genes with signatures of balancing selection were identified as follows: θπ and θw in the upper 25% of the empirical distribution in the descendent population, Tajima's D in the upper 5% of the empirical distribution and *F*_ST_ values <90% of the population pairwise distribution.

To validate genes identified as under selection at the whole gene level, five candidate genes under purifying selection and nine candidate genes under balancing selection were used as input, together with 34 neutral genes, for the mlHKA test (Wright and Charlesworth, 2004). The mlHKA program was run under a neutral model (numselectedloci = 0) and then under a selection model (numselectedloci > 0). Significance was assessed by the mean log likelihood ratio test statistic, where twice the difference in log likelihood between the models is approximately chi‐squared distributed with df equal to the difference in the number of parameters.

To calculate selection at the base pair level, θ_π_, Tajima's D and *F*_ST_ were calculated from CDS sequences of all genes using PopGenome (Pfeifer *et al*., 2014). Criteria to identify sites of purifying selection were as follows: (i) reduction in diversity in the pairwise ancestor/descendant population comparison, greater than the mean gene diversity in the whole genome; (ii) *F*_ST_ > 0; and (iii) negative Tajima's D. Criteria to identify sites of balancing selection were as follows: (i) increase in diversity in the pairwise ancestor/descendant population comparison, greater than the mean gene diversity in the whole genome; (ii) *F*_ST_ > 0; and (iii) positive Tajima's D.

## Supporting information


**Table S1** Sorghum Accessions and their racial and geographic origins.
**Table S2** Fragments per kilobase of exon per million fragments mapped (FPKM) of genes involved in the starch synthesis pathway via the breakdown of D‐fructose from immature grain 16 days post anthesis, sampled at solar midday and midnight. Data bars (red colour) indicate the value of FPKM in relation to the highest expressed gene model.
**Table S3** Fragments per kilobase of exon per million fragments mapped (FPKM) of genes involved in the starch synthesis pathway via the breakdown of α‐D‐glucose from immature grain 16 days post anthesis, sampled at solar midday and midnight. Data bars (red colour) indicate the value of FPKM in relation to the highest expressed gene model.
**Table S4** RNA‐Seq data of Sorghum genes with significant differential expression between solar noon and solar midnight of 16 days post anthesis immature grain.
**Table S5** Nucleotide diversity (θπ), Tajima's D and FST values for the CDS of genes involved in the primary starch synthesis pathway of Sorghum. Values were calculated according to the groups ‘All Lines’, ‘Landraces’ and ‘Wild & Weedy’.
**Table S6** Nucleotide diversity (θπ), Tajima's D and FST values for the CDS of genes involved in the starch synthesis pathway via the breakdown of D‐fructose in Sorghum. Values were calculated according to the groups ‘All Lines’, ‘Landraces’ and ‘Wild & Weedy’.
**Table S7** Nucleotide diversity (θπ), Tajima's D and FST values for the CDS of genes involved in the starch synthesis pathway via the breakdown of α‐D‐glucose in Sorghum. Values were calculated according to the groups ‘All Lines’, ‘Landraces’ and ‘Wild & Weedy’.
**Table S8** Number and type of selection categories scored from flanking starch synthesis pathway genes. Flanking regions included up to ten consecutive gene models both up and down stream of the gene of interest.
**Table S9** Base pairs under the influence of purifying selection from CDS sequence of starch synthesis pathway genes calculated using PopGenome (http://cran.r-project.org/). Symbols correspond to orthologs of maize (*) and rice (#) under selection.
**Table S10** Base pairs under the influence of balancing selection from CDS sequence of starch synthesis pathway genes calculated using PopGenome (http://cran.r-project.org/). Symbols correspond to orthologs of maize (*) and rice (#) under selection.
**Table S11** Maximum‐likelihood analysis of nucleotide polymorphism in a subset of starch synthesis candidate genes for domestication to determine whether a model of neutral or adaptive evolution best explained the patterns of nucleotide polymorphisms. Candidate genes were run seven times with 34 neutral genes used for comparison in each run.

**Method S1** Sampling, RNA extraction and mRNA enrichment for RNA‐Seq analysis.
