# Supplementary material for: Domestication and the storage starch biosynthesis pathway: signatures of selection from a whole sorghum genome sequencing strategy
Source: Plant Biotechnol J. 2016 Jun 11;14(12):2240–53. doi: 10.1111/pbi.12578 (PMC5103234; doi:10.1111/pbi.12578)
Supplement: Supplementary file 2 — Method S1 Sampling, RNA extraction and mRNA enrichment for RNA‐Seq analysis. [file PBI-14-2240-s001.doc]

**Supp. Method 1: Sampling, RNA Extraction and mRNA Enrichment for RNA-Seq Analysis**

Immature grain from the resequenced inbred genotype R931945-2-2 was used for the RNA-Seq experiment. Samples were collected at solar noon, the time at which the sun is at its zenith and solar midnight, twelve hours after solar noon. Samples were then pulverised using a mortar and pestle whilst being kept frozen and until the grain tissue was a fine powder. The pulverised material was then used in the endosperm RNA extraction protocol of Li and Trick (2005). RNA pellets were resuspended in RNAse-free H_2_O and then treated with Turbo DNAse from Ambion (www.thermofisher.com) as per manufacturer’s instructions. To determine the integrity of extracted RNA was analysed on a BioAnalyzer 2100 using a RNA 6000 Nano chip (www.genomics.agilent.com). mRNA capture was then performed through two rounds of DynaBeads oligo dT enrichment (www.invitrogen.com), again using the protocol of the manufacturer. To determine if mRNA-enrichment was successful poly-A treated samples were again run on a BioAnalyzer 2100 and Agilent’s RNA 6000 Pico Chip.

**Ion Torrent PGM RNA-Seq library preparation**

Samples were processed using the published Ion Torrent RNA-Seq protocol (version October 2011). In brief, the following manipulations were performed to the samples. First the RNA was enzymatically fragmented then annealed to Ion Torrent adaptors. cDNA synthesis was performed followed by PCR amplification of adaptor-flanked fragments. The samples were then cleaned up using two rounds of AmbiPure bead purification. Eluted fragments were then quantified using a Qubit 2.0 fluorometer and the dsDNA BR Assay (www.invitrogen.com). Libraries were then diluted as recommended by the Ion Torrent PGM RNA-Seq pipeline.

Diluted libraries were then hybridised onto Ion Torrent Particles using the Ion Torrent One Touch and One Touch ES systems as per manufacturer’s protocols. Fragments captured on enriched Ion Torrent Particles were then sequenced using 200bp Ion Torrent Sequencing chemistry with a combination of 314, 316 and 318 Ion Torrent chips. Sequence data and Q-score determinations were passed through Ion Torrent Suite v2.2.

**RNA-Seq Data Analysis**

The publicly available *S. bicolor* Sbi v2.1 gene set was used as a transcript set alongside the *de novo* transcript assembly as references to quantify gene expression (Paterson *et al.,* 2009). Gene expression was calculated using RSEM v1.2.14 and these counts were passed along to EBSeq v1.5.4 to find differentially expressed genes. All gene expression values were represented via Fragments per kilobase of exon per million fragments mapped (FPKM) (Trapnell *et al.,* 2010). Transcripts, annotations and expression values were matched for all isoforms in both the Sbi v2.1 and *de novo* transcript sets that had FDR <0.05. Comparisons between diurnal/nocturnal samples were also calculated using this pipeline.

**References**

Paterson, A.H., Bowers, J.E., Bruggmann, R., Dubchak, I., Grimwood, J., Gundlach, H., Haberer, G., Hellsten, U., Mitros, T., Poliakov, A., Schmutz, J., Spannagl, M., Tang, H., Wang, X., Wicker, T., Bharti, A.K., Chapman, J., Feltus, F.A., Gowik, U., Grigoriev, I.V., Lyons, E., Maher, C.A., Martis, M., Narechania, A., Otillar, R.P., Penning, B.W., Salamov, A.A., Wang, Y., Zhang, L., Carpita, N.C., Freeling, M., Gingle, A.R., Hash, C.T., Keller, B., Klein, P., Kresovich, S., McCann, M.C., Ming, R., Peterson, D.G., Mehboob-ur-Rahman, Ware, D., Westhoff, P., Mayer, K.F., Messing, J., and Rokhsar, D.S. (2009) The *Sorghum bicolor* genome and the diversification of grasses. *Nature*, **457**, 551-556.

Trapnell, C., Williams, B., Pertea, G., Mortazavi, A., Kwan, G., van Baren, M., Salzberg, S., Wold, B., and Pachter, L. (2010) Transcript assembly and quantification by RNA-Seq reveals unannotated transcripts and isoform switching during cell differentiation. *Nat. Biotechnol*. **28**, 511-515.
